# Epidemiological profile and temporal trend of hospitalizations for Alzheimer's disease in the Brazilian Unified Health System (2012–2022)

**DOI:** 10.1055/s-0045-1813643

**Published:** 2026-01-25

**Authors:** Eduarda Bertella, Franklin Alberto Asanza Correa, Rodrigo da Rosa Iop, Nazaré Otília Nazario, Franciele Cascaes da Silva, Luana Meneghini Belmonte

**Affiliations:** 1 Universidade do Sul de Santa Catarina, Curso de Medicina, Palhoça SC, Brazil.; 2 Universidade do Sul de Santa Catarina, Núcleo de Epidemiologia e Pesquisa, Florianópolis SC, Brazil.

**Keywords:** Alzheimer Disease, Health Profile, Dementia, Aged, Hospitalization

## Abstract

**Background:**

Alzheimer's disease (AD) is a progressive neurodegenerative condition causing cognitive decline. Given the aging population and increasing prevalence, understanding hospital morbidity patterns is crucial for improving prevention strategies and public health planning.

**Objective:**

To analyze the epidemiological profile and temporal trend of AD hospitalizations in Brazil between 2012 and 2022.

**Methods:**

The present mixed ecological study used data from the Hospital Information System of the Unified Health system. We calculated the frequencies for sex, age group, and skin color, along with the average length of stay and total costs. Simple linear regression was used for the temporal trend analysis across sex, age groups by sex, and regions.

**Results:**

A higher proportion of hospitalizations was observed in females (65.80%), individuals aged 80 years or older (60.12%), and white individuals (49.06%). The average hospital stay was 21.6 days, with a total cost of R$ 23,306,587.01. The hospitalization trend was stable across both sexes, all age groups, and most regions (4.88/100 thousand hospitalizations), except for the Northeast, which showed a significant increase (β = 0.408; *p* < 0.001).

**Conclusion:**

The profile of AD hospitalizations in Brazil is predominantly female, in individuals over 80 and white. While the trend remained stable nationally, there was an increase in the Northeast region. Additionally, a reduction in the average number of hospitalization days and hospital costs was observed throughout the period analyzed.

## INTRODUCTION

Alzheimer's disease (AD) is a neurodegenerative disorder characterized by the insidious and gradual loss of cognitive functions, with an impact on activities of daily living and neuropsychiatric symptoms, 1 while dementia is a clinical syndrome marked by symptoms that affect areas of the brain involved in memory, thinking, behavior, and emotion. 2 The most common comorbidity related to dementia is AD, responsible for 60 to 80% of cases, constituting an important cause of morbidity and mortality in the elderly. 3

Most cases of AD are caused by an interaction between genetic, environmental, and lifestyle factors. Nevertheless, the hypothesis of amyloid protein deposition remains the main characteristic finding, despite growing evidence that the disease develops in a multifactorial manner. The main risk factor for AD is aging, since the prevalence in the 65-to-69 age group is 2%; at 85 years or older, it increases to 23.4%, and above 90, it is 36%. 4 In addition, there is an exponential increase in incidence and prevalence with age, doubling every 5 years for individuals over 65. 5 Women are almost twice as likely to develop the disease, and although longer life expectancy contributes to this difference, the exact causes are still unknown. 6 7

According to the World Health Organization, dementia is now classified as the seventh leading cause of death worldwide; therefore it is an important issue to be addressed by public health. 8 Despite this, the percentage of underdiagnosed cases of dementia is still high, even in countries with well-developed economies. 9 According to Gauthier et al., around 75% of dementia cases worldwide are undiagnosed; 10 in Brazil, the estimate is similar, at around 70%. 11

A study based on Medicare data between 1999 and 2014 revealed that patients with AD were more likely to experience emergencies from all causes, visits to the emergency room, hospitalizations for various reasons, and falls. 12 Thus, it is crucial to distinguish whether hospitalizations and emergencies are caused directly by AD or by other conditions that may coexist with the disease, to better understand the direct impact of the disease and develop more effective intervention strategies.

The population projection, calculated by Instituto Brasileiro de Geografia e Estatística (IBGE, Brazilian Institute of Geography and Statistics), predicts that the elderly will make up to 28.44% of the Brazilian population in 2050, while in 2022 they represented 15.16%. 13 This projection reflects that the country is following a trend of population aging and, consequently, diseases associated with the elderly will become a relevant public health problem. According to Alzheimer's Disease International, there were more than 55 million cases of dementia in 2020, and this number is estimated to reach 139 million in 2050, 14 reinforcing the need to understand the epidemiology of AD.

Analysis of the costs and length of hospital stays for AD in Brazil in previous studies has revealed a substantial economic impact, with long hospitalization times and significant expenses. 15 16 Given the aging population and the growing prevalence of the disease, obtaining updated data on hospital morbidity patterns is essential for planning effective public policies. However, the literature lacks a recent and comprehensive epidemiological overview, especially regarding regional variations and the economic impact over time. To address this gap, the present study employed a robust methodology, using data from the Hospital Information System of the Brazilian Unified Health System (Sistema de Informações Hospitalares do Sistema Único de Saúde [SIH-SUS], in Portuguese), a reliable national source covering public hospitalizations for AD in the country.

Thus, the current study aimed to analyze the epidemiological profile and temporal trend of hospitalizations for AD in Brazil between 2012 and 2022. The results can assist health managers in decision-making, contributing to the development of specific public policies and the definition of prevention plans, prioritizing the population most vulnerable to the disease, contributing to quality of life, and mitigating the costs of these processes.

## METHODS

### Study design

The present is a mixed ecological study (profile and time series), conducted using data obtained from SIH-SUS, managed by the Department of Computer Science of the Unified Health System (Departamento de Informática do Sistema Único de Saúde [DATASUS], in Portuguese). 17 The study included data on hospitalizations of elderly patients between 2012 and 2022 diagnosed with AD according to the 10th Revision of the International Classification of Diseases (ICD-10) 18 G30.0–G30.9, available in the SIH-SUS, of both sexes and age groups from 60 years old, from all regions of Brazil. There were no exclusion criteria.

### Data collection

Data extraction was performed using the TABWIN software (DATASUS), in comma-separated values (cvs) format. Information on the population was taken from the latest Brazilian population projection by IBGE (available at: https://www.ibge.gov.br/estatisticas/sociais/populacao/9109 - projecao-da-populacao.html). 13 Subsequently, the data were exported to a Microsoft Excel (Microsoft Corp.) spreadsheet for the calculation of rates.

### Statistical analysis

For the profile analysis, the simple frequency (n) and relative frequency (%) of the following variables were calculated: gender, age group, and skin color, information regarding the length of stay (days) and costs (in reais), obtained from the SIH-SUS itself. For the trend analysis, the independent variable was the year in which the information was recorded (2012–2022), while the dependent variables included gender (male and female); age group by gender (60–69, 70–79, 80 or over), and regions of Brazil (North, Northeast, Midwest, Southeast, and South). The overall hospitalization rate was calculated using the ratio between the number of hospitalizations per AD and the total population of Brazil, multiplied by 100 thousand inhabitants. For each year of the period, the specific hospitalization rate was calculated by dividing the number of hospitalizations due to AD according to sex, age group/sex, regions, and the reference population in the period per year, multiplied by 100 thousand inhabitants.

After calculating the rates, the temporal trend analysis was performed with the IBM SPSS Statistics for Windows (IBM Corp.) software, version 20.0., using the simple linear regression method, according to the formula Y = b0 + b1X, where Y = standardized coefficient, b0 = average coefficient for the period, b1 = average annual increase, and X = year. To examine the behavior (increase, decrease, or stability) of the morbidity coefficient, the value (positive or negative) and statistical significance of the regression coefficient (β) were evaluated. The statistical significance of the model was confirmed for a value of *p* < 0.05.

### Ethical considerations

The study complied with the ethical precepts of the National Health Council, in its Resolutions No. 466/2012 and 510/2016, and, as it involved secondary data in the public domain, evaluation by the research ethics committee was not necessary.

## RESULTS

Data from 14,496 hospitalizations due to AD between 2012 and 2022 in Brazil were analyzed. Table 1 shows the profile of hospitalizations for AD according to gender, age group, and skin color. Throughout the period analyzed, a higher proportion of hospitalizations was observed among females (65.80%), those aged 80 years or older (60.12%), and those with white skin color (49.06%).

**Table 1:** Profile of hospitalizations for Alzheimer's disease in Brazil between 2012 and 2022, by sex, age group and skin color

Variables		n	%
**Sex**	Male	4,957	34.20
	Female	9,539	65.80
**Age group**	60–69 years	1,380	9.52
	70–79 years	4,401	30.36
	80 years or older	8,715	60.12
**Skin color**	White	7,112	49.06
	Black	560	3.86
	Brown	2,774	19.14
	Yellow	171	1.18
	Indigenous	2	0.01
	Without information	3,877	26.75

Table 2 shows the average length of hospital stay and associated costs, as well as a description of the total number of hospitalizations per year. During the period analyzed, there was a reduction in the average number of days of hospitalization, with the exception of 2019. The lowest average number of days of hospitalization was 13.2 days (2022), while the highest was 31.5 days (2012), with a reduction of 18.3 days in hospital stays. The cost associated with hospital admission was higher between 2012 and 2019, exceeding R$ 2 million, decreasing from 2020 onwards, with a reduction of 26.35%.

**Table 2:** Average days of hospitalization (hospital stay) and associated costs for Alzheimer's disease in Brazil between 2012 and 2022

Year	Number of hospitalizations	Average hospital stay (days)	Cost (reais)
2012	879	31.5	2,126,100.54
2013	953	31.3	2,270,911.00
2014	1,151	31.6	2,860,188.97
2015	1,536	22.4	2,378,838.97
2016	1,457	22.5	2,403,828.55
2017	1,516	17.0	2,081,511.30
2018	1,503	17.6	2,031,018.12
2019	1,552	19.6	2,502,789.75
2020	1,179	16,3	1,595,813.92
2021	1,253	15,0	1,582,721.42
2022	1,572	13,2	1,472,864.47

The analysis of the temporal trend showed stability in the rates of hospital admissions for AD in Brazil during the period analyzed (β = −0.006; *p* = 0.944) ( Table 3 ). There was a trend toward stability in both sexes ( *p* > 0.005), with a higher average hospital admission rate among females (5.75/100 thousand inhabitants). A temporal trend toward stability was observed in all age groups for both males and females ( *p* > 0.05). The highest average hospitalization rate was in the 70-to-79 years age group for males (15.77/100 thousand inhabitants) and in the 80+ age group for females (22.88/100 thousand inhabitants). Regarding Brazilian regions, only the Northeast region showed an upward trend during the period (β = 0.408; *p* < 0.001) with an average rate of 3.93/100 thousand inhabitants.

**Table 3:** Temporal trend of hospitalizations for Alzheimer's disease in Brazil, from 2012 to 2022, according to sex, age group by sex and regions

		Average rate	ß (‡)	IC95%	*p* -value	Trend
**General rate**		4.88	0.006	−0.176; 0.187	0.944	–
**Sex**	Male	3.79	−0.024	−0.161; 0.113	0.704	–
	Female	5.75	0.028	−0.190; 0.247	0.667	–
**Age group male**	60–69 years	0.79	−0.015	−0.051; 0.020	0.355	–
	70–79 years	15.77	0.004	−0.646; 0.653	0.990	–
	≥ 80 years	15.63	−0.129	−0.806; 0.548	0.677	–
**Age group female**	60–69 years	0.87	0.004	−0.047; 0.054	0.874	–
	70–79 years	5.31	−0.076	−0.298; 0.146	0.457	–
	≥ 80 years	22.88	0.234	−0.607; 1.074	0.545	–
**Regions**	North	5.02	0.077	−0.103; 0.257	0.358	–
	Northeast	3.93	0.408	0.250; 0.566	<0.001	↑
	Southeast	10.26	−0.280	−0.842; 0.282	0.288	–
	South	13.21	0.016	−0.220; 0.252	0.881	–
	Midwest	7.75	0.392	−0.044; 0.828	0.072	–

Notes: ß (‡) - linear regression coefficient; ↑ Increasing; ↓ Decreasing; - Stability.

## DISCUSSION

In the present study, women accounted for 65.80% of hospitalizations for AD (AD) in all years evaluated, as did patients aged 80 years or older (60.12%) and white individuals (49.06%). The average length of stay was 21.6 days, with an average cost of R$ 2,118,780.63/year. Hospitalizations remained stable between 2012 and 2022, except in the Northeast region, where an increase was recorded. Despite this stability, hospitalization rates increased with age, especially among elderly people over 80 years of age and women.

In this sense, regarding the profile of hospitalizations for AD according to gender, Sousa et al., 19 in a descriptive observational study that analyzed SIH-SUS data between 2018 and 2022, and Zalli et al. 20 in a cross-sectional study between 2008 and 2015, showed a higher prevalence of hospitalizations for dementia in males (51.57% and 52.4%, respectively), including AD, vascular dementia, and unspecified dementias. In the present study, which analyzed AD exclusively, a higher prevalence of hospitalizations was observed in females.

The studies by Piovesan et al., 21 Santos et al., 22 and Silva et al. 23 confirm our findings, highlighting females as having the highest frequency of hospitalization, with 64.80% (2010–2020), 64.54% (2008–2018) and 63.40% (2013–2017), respectively. Given this, it can be inferred that the higher prevalence of hospitalizations in women is mainly explained by their greater longevity, which increases the risk of AD. In addition, the drop in estrogen during menopause may be related to a higher neurodegenerative risk due to the reduction in neuroprotection caused by hormonal transition. 24 Another hypothesis is low female education levels, 25 26 but the change in the educational profile among women makes it essential to conduct studies to monitor the epidemiological evolution of the disease.

In addition, the aforementioned authors 21 22 23 confirm the findings of this study regarding age profile, demonstrating a higher prevalence of hospitalizations in patients aged 80 years or older. Advanced age, the main risk factor for AD, increases physical and cognitive frailty and is associated with comorbidities such as hypertension and diabetes, which increase the need for hospitalizations in this age group, justifying the predominance of hospitalizations. 4 5 12

Furthermore, studies 21 22 23 show a higher prevalence of hospitalizations for AD among white people, but the findings of Santos et al. 22 and Silva et al. 23 stand out, who recorded a high frequency of cases without specified skin color or race (29.07% and 25%, respectively), which is consistent with our results (26.74% without information). Skin color may be an indirect risk factor, as the higher prevalence among white people may reflect better access to diagnosis, while the underrepresentation of brown and black people suggests difficulties in accessing medical care. 27 28 29 It is worth noting that cases with unspecified skin color constitute a limitation of the study, as they represent a significant portion of hospitalizations, which could impact the epidemiological outcomes of the disease.

Between 2012 and 2022, there was a reduction in the average length of hospital stay for AD from 31.5 to 13.2 days, in addition to a 26.35% drop in hospital costs. In this context, Santos et al. 22 and Silva et al. 15 presented longer average stays (27.4 and 28.61 days, respectively), but it is impossible to say whether the results of the current study reflect improvements in hospital care or early discharges due to deaths. Regarding associated costs, an ecological time series study by Silva et al. 15 pointed out that, between 2008 and 2020, the total cost of AD health care was R$27,617,699.74, which shows a positive reduction for public health, despite the growing number of cases of the disease. On the other hand, an American study by Wong 30 estimated that the costs of AD reached US$305 billion in 2020, with a projected growth, a relevant scenario for Brazil, which faces an aging population and an increase in chronic degenerative diseases.

The present study showed stability in the rates of hospitalizations for AD in Brazil, with a higher prevalence among women (5.75/100 thousand) and elderly people over 80 years of age (22.88/100 thousand). The Northeast region was the only variable with an upward trend (β = 0.408; *p* < 0.001), despite having the lowest average rate (3.93/100 thousand) among the regions. Regarding the trend in hospitalizations, a study by Feter et al. 31 indicated an 87.7% increase in hospitalizations between 2010 and 2019 and a 127% increase in the proportion of people with AD since 1990. In addition, Silva et al. 15 indicated an increasing trend in hospitalizations between 2008 and 2020, differing from the findings of the present study, possibly due to the difference in the period analyzed.

Regarding gender, despite the stability in hospitalizations, women had higher average rates both overall (5.75/100 thousand) and in the 60-to-69 age group (0.87/100 thousand) and over 80 age group (22.88/100 thousand). A time series study by Araújo et al. 16 showed that 9,175 (65%) hospitalizations occurred in women. According to Garre-Olmo, 7 the probability of a person developing AD over their lifetime is ∼ 1 in 5 women and 1 in 10 men. In the study conducted by Feter et al., 31 there was a marked increase in hospitalizations for AD among people aged 80 years or older (115.1%), probably due to the aging of the population and the higher prevalence of the disease in the elderly. In a tertiary hospital, hospitalizations of patients with AD and Parkinson's disease (PD) were compared, and it was found that patients with AD were, on average, older and had higher rates of rehospitalization and in-hospital mortality, highlighting advanced age as a risk factor. 32

Hospitalization rates for AD were higher in the Northeast and Midwest, with increases of 172.1% and 144.2%, respectively, in these regions, despite lower absolute numbers. In addition, the authors state that, compared with other chronic diseases, AD showed the largest increase in absolute numbers and hospitalization rates in Brazil during the study period. 31 Other evidence 22 33 shows that 57.22% to 59% of hospitalizations occurred in the Southeast. In Silva et al.'s 15 ecological study, an increasing trend was observed in all regions of Brazil between 2008 and 2020, with the highest annual percentage variation in the Northeast (3.57). One possible explanation for the increasing trend in the Northeast would be improved access to health services. On the other hand, the higher rates found in the South and Southeast can be attributed to the higher population concentration in these regions. However, the variations between the analyses highlight the need for more comparative research on the geographical distribution of the disease in Brazil.

Analyzing the limitations of the study, it is crucial to note that the results may be underestimated. The main reason is the exclusion of data from the private sector, since the analysis was based exclusively on the SIH-SUS. This approach, while allowing a detailed view of the public system, does not reflect the complete picture of AD in Brazil, where many diagnoses and hospitalizations occur in private hospitals.

In addition, the nature of secondary data presents other limitations, such as the lack of standardization in the collection of information and hospitalization records. Furthermore, the heterogeneity of Brazilian regions, with their distinct demographic characteristics and access to health services, may have impacted the uniformity of the results obtained.

Finally, it was concluded that, between 2012 and 2022, the profile of hospitalizations for AD in Brazil consisted of women; patients aged 80 years or older; and white individuals. Hospitalizations for AD remained generally stable during the period, with a tendency toward stability according to gender, age group, and Brazilian region, except for the Northeast, which showed an upward trend. There was also a decrease in the average length of stay and hospital costs over the period analyzed.

Epidemiological studies play a crucial role in the formulation of public health policies, especially in conditions such as AD, whose etiology is still partially unknown, offering valuable information for understanding the processes underlying the disease. It is worth emphasizing again that the aging of the Brazilian population presents significant challenges, with a significant increase in cases of AD and a growing demand for hospital services, reinforcing the need for more comprehensive research to inform health management in the country.
